# Ancient Host-Virus Gene Transfer Hints at a Diverse Pre-LECA Virosphere

**DOI:** 10.1007/s00239-025-10246-8

**Published:** 2025-04-29

**Authors:** Sangita Karki, Zachary K. Barth, Frank O. Aylward

**Affiliations:** 1 Department of Biological Sciences, Virginia Tech, 926 West Campus Drive, Blacksburg, VA 24061 USA; 2 Center for Emerging, Zoonotic, and Arthropod-Borne Infectious Disease, Virginia Tech, Blacksburg, VA 24061 USA

**Keywords:** Giant viruses, Mirusviruses, Virus factory, Early eukaryotes, Nucleocytoviricota

## Abstract

The details surrounding the early evolution of eukaryotes and their viruses are largely unknown. Several key enzymes involved in DNA synthesis and transcription are shared between eukaryotes and large DNA viruses in the phylum *Nucleocytoviricota*, but the evolutionary relationships between these genes remain unclear. In particular, previous studies of eukaryotic DNA and RNA polymerases often show deep-branching clades of eukaryotes and viruses indicative of ancient gene exchange. Here, we performed updated phylogenetic analysis of eukaryotic and viral family B DNA polymerases, multimeric RNA polymerases, and mRNA-capping enzymes to explore their evolutionary relationships. Our results show that viral enzymes form clades that are typically adjacent to eukaryotes, suggesting that they originate prior to the emergence of the Last Eukaryotic Common Ancestor (LECA). The machinery for viral DNA replication, transcription, and mRNA capping are all key processes needed for the maintenance of virus factories, which are complex structures formed by many nucleocytoviruses during infection, indicating that viruses capable of making these structures are ancient. These findings hint at a diverse and complex pre-LECA virosphere and indicate that large DNA viruses may encode proteins that are relics of extinct proto-eukaryotic lineages.

**Supplementary Information:**

The online version contains supplementary material available at 10.1007/s00239-025-10246-8.

## Introduction

Members of the phylum *Nucleocytoviricota* comprise a broad range of large dsDNA viruses that infect both multicellular and unicellular eukaryotic hosts (Aylward et al. 2021). Families within the *Nucleocytoviricota* include the *Poxviridae*, *Asfarviridae,* and *Iridoviridae*, which comprise metazoan viruses that have been the subject of intense study for decades, as well as families that infect primarily algae and protists, such as the *Mimiviridae, Phycodnaviridae,* and *Marseilleviridae* (Bosmon et al. 2025). Nucleocytoviruses are found in a wide range of habitats, and metagenomic studies have recently recovered diverse lineages within this phylum that have not yet been cultivated (Mihara et al. 2018; Schulz et al. 2018; Karki et al. 2021; Farzad et al. 2022). The genomes of nucleocytoviruses often reach > 500 kbp and encode hundreds of genes, and some even reach lengths of up to 2.7 Mbp. Early comparative genomic studies that first demarcated the *Nucleocytoviricota* (then referred to as Nucleo-Cytoplasmic Large DNA viruses, or NCLDV) identified a set of core genes involved in DNA replication and repair, transcription, and some other core functions, and used this as evidence of an ancient shared evolutionary history that unites viruses in this lineage (Iyer et al. 2001, 2006). Subsequent comparative genomic studies have provided further evidence that nucleocytoviruses emerged from smaller viruses and underwent periods of subsequent genome expansion due to gene duplication and acquisition from their hosts (Filée and Chandler 2010; Koonin and Yutin 2018, 2019), though the timing of these events has remained enigmatic.

Nucleocytoviruses have frequently exchanged genes with eukaryotes over their long co-evolutionary history. As a result, many nucleocytovirus lineages have acquired a range of genes from their hosts and, thereby, encode numerous cellular hallmark genes that are common in eukaryotes but rare or absent from other viral lineages (Moniruzzaman et al. 2020a, 2023; Brahim Belhaouari et al. 2022). These include viral genes involved in translation, DNA replication and repair, central carbon metabolism, cytoskeletal structure, and others. Although the timing and direction of gene transfer can be difficult to ascertain, recent viral acquisition of host genes involved in nutrient transport, phototaxis, and sphingolipid metabolism has been reported (Monier et al. 2009, 2017; Rozenberg et al. 2020). Other core viral genes, such as DNA and RNA polymerase subunits and tRNA synthetases, have existed in the *Nucleocytoviricota* for longer periods of time and their evolutionary links to eukaryotic homologs is less clear (Takemura et al. 2015; Yoshikawa et al. 2019; Guglielmini et al. 2019; Kijima et al. 2024). Although host-to-virus gene transfer is typically thought to be more common, many endogenous nucleocytoviruses can be found in eukaryotic genomes, providing a mechanism for virus-to-host transfer (Filée 2014; Moniruzzaman et al. 2020b; Zhao et al. 2023; Sarre et al. 2024). Studies focused on multi-subunit RNA polymerase (Guglielmini et al. 2019), DNA topoisomerase IIA (Guglielmini et al. 2022), and actin (Cunha et al. 2022) have proposed ancient virus-to-eukaryote gene transfers, though it remains difficult to rule out alternative scenarios.

The evolution of eukaryotes represents a major evolutionary transition in the evolution of life on Earth (Gabaldón 2021) and yet the details of this process remain a riddle (Koonin et al. 2015; Richards et al. 2024). Many complex features of eukaryotes emerged in the stem eukaryotic lineage prior to the emergence of LECA, but the order in which these traits emerged is debated. Given the possible pre-LECA origin of nucleocytoviruses, it has been proposed that the co-evolution of these viruses and their hosts played a role in eukaryogenesis (Forterre and Gaïa 2016). Many nucleocytoviruses encode genes involved in DNA synthesis, transcription, and mRNA capping that are shared with eukaryotes and are required for the maintenance of complex viral factories, transient organelles that are formed during infection to replicate viral genomes and package virions. In this study, we sought to examine the evolutionary links between eukaryotes and nucleocytoviruses through in-depth phylogenetic examination of these enzymes. Our results provide insights into the origin of nucleocytoviruses, and lead us to hypothesize that extant viral genomes may harbor relics of proto-eukaryotic lineages that have since gone extinct.

## Results and Discussion

To shed light on the evolutionary origins of the eukaryotic replisome components, we performed phylogenetic analysis of both cellular and viral proteins involved in this process. Eukaryotic genome replication is a complex process that is performed by a suite of DNA polymerases and accessory factors. Eukaryotes encode four family B DNA polymerases (PolBs), and molecular studies have shown that Polε and Polδ perform the majority of leading and lagging strand synthesis (Burgers and Kunkel 2017; Kazlauskas et al. 2020). The two other PolBs—Polα and Polζ—have major roles in replication initiation and DNA repair, respectively. The DNA sliding clamp—also called the Proliferating Cell Nuclear Antigen, or PCNA—is a key component of the replisome that associates with both *δ* and *ε* polymerases and prevents them from dissociating from DNA during polymerization, effectively providing the processivity that is needed for replication of large cellular genomes (Burgers and Kunkel 2017).

**Fig. 1:**
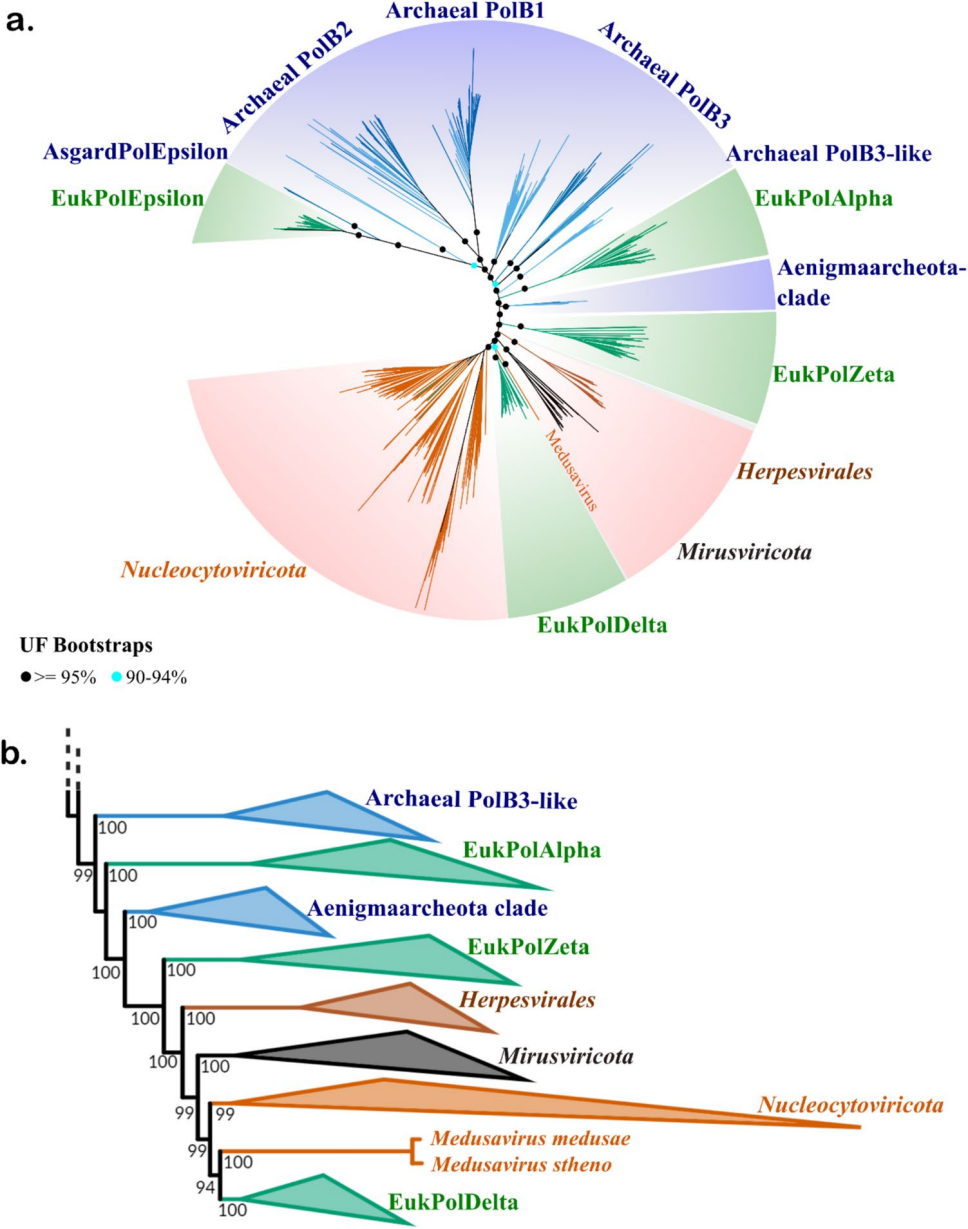
Phylogenetic tree of DNA polymerase family B demonstrating nested placement of Polδ in a viral clade and Polε with Asgard archaea (957 total sequences, 1417 sites). **a** Maximum-likelihood analysis was performed using IQ-TREE using the complex model LG+C60+F+G. Ultrafast bootstrap support values for select deep-branching nodes are shown (black dot > = 95%, blue dot 90–94%). For clarity, support values are only provided for select internal nodes. **b** Rectangular representation of the region of the polB phylogenetic tree highlighting the evolutionary relationships between viral groups and eukaryotic Polδ. Values at nodes represent their ultrafast bootstrap support. Polζ = PolZeta; Polα = PolAlpha; Polδ = PolDelta and Polε = PolEpsilon in the figure (Color figure online)

For the PolB phylogeny, we included as broad a sampling of enzymes as possible in order to provide an accurate reconstruction of ancient evolutionary events. We included PolBs from eukaryotes and archaea, as well as several distinct lineages of large DNA viruses, such as herpesviruses (order *Herpesvirales*), giant viruses (phylum *Nucleocytoviricota*), and members of the recently discovered mirusvirus lineage (Gaïa et al. 2023). For multi-sequence alignment, we used the Muscle5 program, which has been shown to substantially improve the alignment of divergent proteins (Edgar 2022). To assess the quality of tree topology, we employed both regular and complex substitution models in our phylogenetic analyses (LG+F+R10 and LG+C60+F+G, respectively), we employed different levels of taxon sampling (Fig. 1a and b and Supplementary Fig. 3a), and we performed phylogenetic reconstruction on alignments that were trimmed to several different levels of stringency (Supplementary Fig. 7). In our resulting trees, we found that Polδ formed a distinct clade sister to most of the *Nucleocytoviricota* and nested within a broader clade that includes the herpesviruses and mirusviruses (Fig. 1). Medusavirus PolBs were placed basal to the nucleocytovirus/Polδ clade, consistent with previous findings that these viruses encode a distinct variant of this enzyme (Yoshikawa et al. 2019). These results were well supported in all of the trees that we constructed (> 99% ultrafast bootstrap support in all cases). These trees also showed Polε clustered near a small clade of Asgard archaeal PolB homologs, mostly belonging to members of the Hodarchaeota and Heimdallarchaeota (Supplementary Table 2). Importantly, Polε is a large enzyme that appears to have been formed through the ancient fusion of two distinct PolB enzymes, with only the N-terminal domain retaining catalytic activity (Tahirov et al. 2009). In our alignment only the N-terminal PolB domain was aligned with homologous PolB sequences, suggesting that this domain in eukaryotes was derived from Asgard archaea.

Fast-evolving sites may introduce noise and obscure phylogenetic inference in protein families (Brinkmann et al. 2005), and so to further confirm the topology we made multiple sets of PolB trees in which increasing numbers of fast-evolving sites were iteratively removed from the alignment (see Methods for details). The overall topology of these trees remained consistent until 40% of all alignment positions were removed, after which the overall quality of the tree deteriorated as evidenced by the collapse of monophyly in major clades of archaea and viruses (Supplementary Fig. 4a). This analysis provides another confirmation of the deep-branching placement of both eukaryotic and nucleocytovirus clades adjacent to each other. Overall, this finding is consistent with earlier studies that have found phylogenetic affinity between eukaryotic and viral PolBs in the Polδ clade (Takemura et al. 2015; Yoshikawa et al. 2019).

The sliding clamp (PCNA) associates with both polymerases δ and ε during DNA replication and is a key component of processive replication that is needed for whole-genome synthesis. Due to the key role of the DNA sliding clamp in replication processivity, we also performed phylogenetic analyses on viral and cellular homologs of this protein, using methods similar to those that we employed for the PolB phylogenies (see Methods and supplementary figures, Fig. 1). The robustness of the PCNA tree is worse than that of the PolB tree, likely because of the shorter length of this protein (mean length of ~ 270 aa for PCNA compared to > 1000 aa for most PolBs). It is not possible to make any conclusions based on the PCNA tree, although the eukaryotic PCNA once again is placed near viral homologs, suggesting that it may have an evolutionary history similar to that of Polδ.

**Fig. 2:**
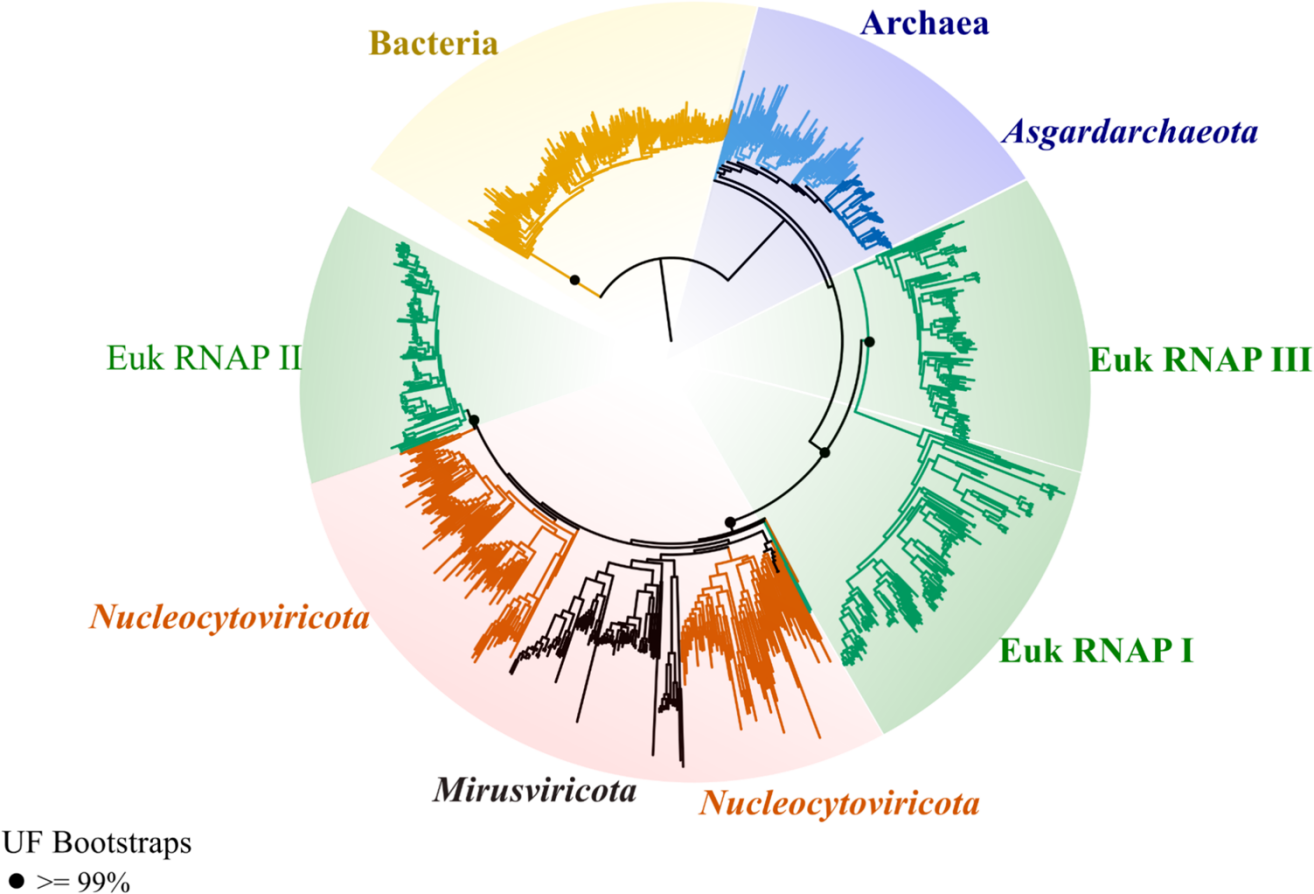
Phylogenetic tree for RNA Polymerase (RNAP). The alignment is based on a concatenated set of Beta and Beta prime subunits from 1017 sequences (resulting in a total alignment length of 3812 sites). Maximum-likelihood analysis was performed using IQ-TREE under a complex model (LG+C60+F+G). The dots on the branches represent ultrafast bootstrap support values (black dot > = 99%). For clarity, support values are only provided for selected internal nodes. Full trees are available in the supplemental material. The tree is rooted between the bacteria and all other taxa (Color figure online)

To examine the evolutionary relationships between viral and eukaryotic RNA polymerases, we then examined trees of multimeric RNA polymerase (RNAP). RNAP is a key enzyme in which the two major subunits are found in a single copy in bacteria, archaea, and some DNA viruses, and three copies in eukaryotes (referred to as I, II, and III) (Werner and Grohmann 2011). A previous phylogenetic analysis of eukaryotic and viral RNAP found that viral enzymes tended to cluster near eukaryotic RNAP II (Guglielmini et al. 2019). We examined the evolution of this enzyme using an updated genomic representation of both viral and cellular proteins, including those from the recently discovered mirusviruses. Using a complex substitution model, our results suggest that nucleocytoviruses place near eukaryotic RNAP II (LG+C60+F+G; 100% bootstrap support), but that eukaryotic RNAP I and III form deep-branching groups (Fig. 2). Similar to our PolB analyses, we confirmed this result with extensive testing of alternative phylogenetic models, alignment trimming severity, and taxon sampling (see Methods). It is notable that RNAP II is responsible for mRNA transcription in eukaryotes, which is similar to the role that this enzyme plays in nucleocytoviruses.

In our analysis, RNAP I and RNAP III, which are involved primarily in rRNA and tRNA transcription, form clades that are distinct and basal branching to RNAP II. With the exception of RNAP III from *Spironucleus salmonicida* and RNAP II from *Giardia intestinalis*, which formed long branches that were placed basal to the viral/RNAPII clade, the different RNAP classes formed distinct clades. It is notable that the branches leading to the diversification of RNAP I, RNAP III, and viral/eukaryotic RNAP II are extremely short, indicative of a rapid evolutionary transition that occurred at or around the time of eukaryogenesis. It is possible that this may have been caused by several rapid duplication and divergence events in their proto-eukaryotic ancestors. To potentially resolve the branching order of RNAP I, II, and III, we constructed a series of RNAP trees in which a range of fast-evolving sites were removed, but these additional trees did not provide strong support for any particular scenario of duplication and divergence (Supplementary Fig. 4b). This is perhaps not surprising, given that resolving the branching order of ancient evolutionary events that occurred close together in time is notoriously difficult (Salichos and Rokas 2013).

**Fig. 3:**
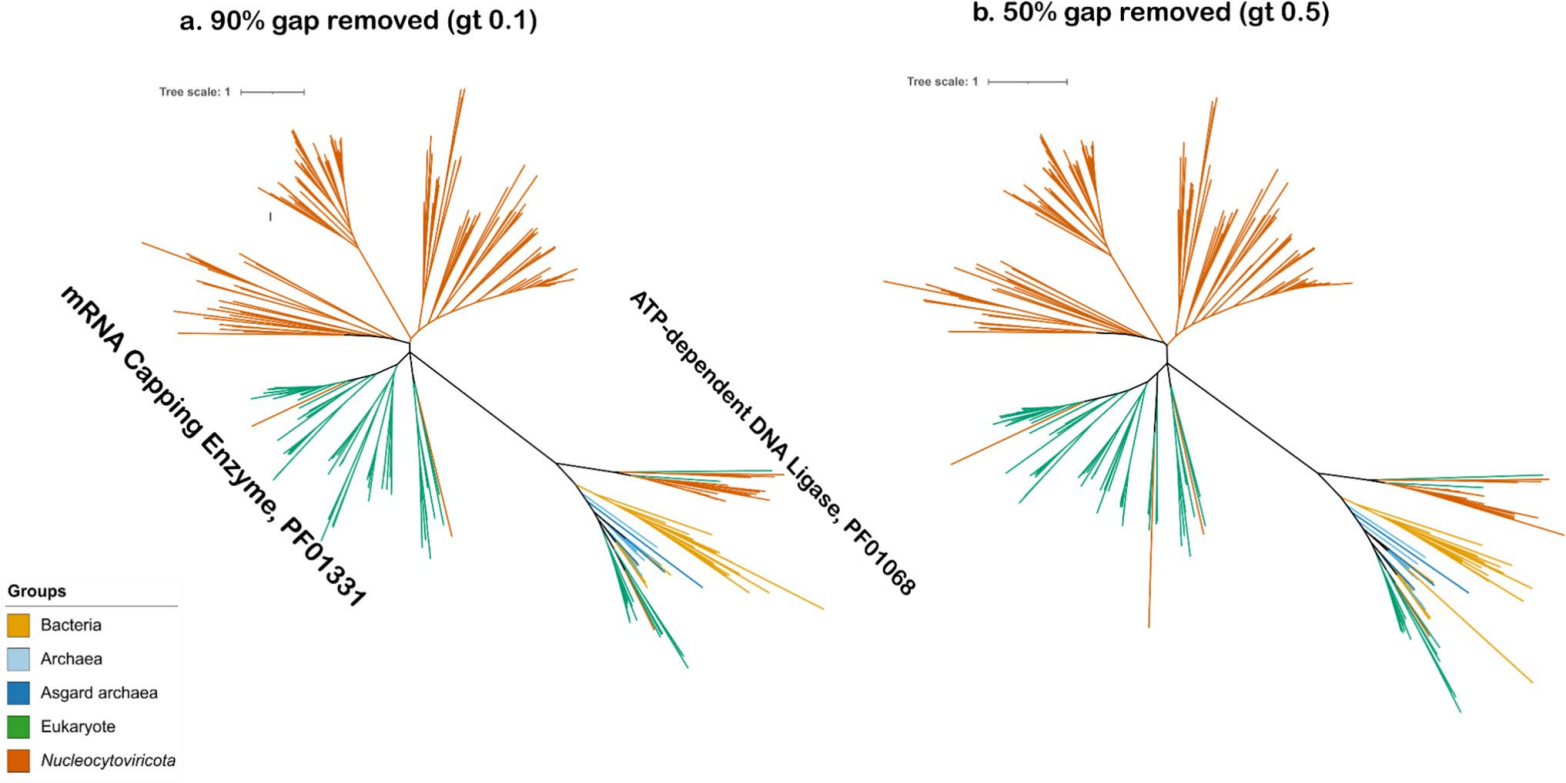
Phylogenetic tree for mRNA-capping enzyme (upper clade) along with ATP-dependent DNA ligase (lower clade, labeled). Trees were made using different trimming strategies. Sites with 90% gaps removed (left) resulting in total alignment of 856 sites. Sites with 50% gaps removed, resulting in total alignment of 326 sites (right). Maximum-likelihood analysis was performed using IQ-TREE using LG+F+R10 model

Lastly, we performed a phylogenetic analysis of eukaryotic and viral mRNA-capping enzymes. The phylogenetic analysis of mRNA-capping enzyme (PF01331) and ATP-dependent DNA ligase (PF01068) across eukaryotes and nucleocytoviruses revealed distinct clades corresponding to groups of eukaryotic and viral enzymes (Fig. 3). Three nucleocytovirus mRNA-capping enzymes belonging to the Red seabream iridovirus, Three spot gourami iridovirus, and a metagenome-derived mimivirus (SRX327520.21) exhibited long branches that clustered with eukaryotes, suggesting possible secondary transfers from eukaryotes to viruses. Two different gap trimming thresholds were applied: 90% (-gt 0.1 in trimAl) and 50% (-gt 0.5 in trimAl), allowing us to assess the impact of alignment stringency on tree topology. Under both conditions, the mRNA-capping enzyme clade consistently showed placement of nucleocytoviruses adjacent to eukaryotes, suggesting an ancient evolutionary relationship among these lineages. For the ATP-dependent DNA ligase, the *Nucleocytoviricota* clades seems to be a paraphyletic with *Pokkesviricetes* clades clustering within the eukaryotes, adjacent to the bacterial-archeal clades while the *Megaviricetes* formed a different clade. This suggests that the ATP-dependent DNA ligases were acquired by the different viral lineages at different evolutionary times, as previously suggested by (Yutin and Koonin 2009).

## Conclusions

Assessing the evolutionary origins of viruses had traditionally been challenging owing to their fast evolutionary rates coupled with their paucity of useful phylogenetic marker genes (Koonin et al. 2022; Aylward and Moniruzzaman 2022). Moreover, assessing the emergence of viral lineages relative to cellular diversity is further complicated by the limited number of viral genes that are shared with cellular groups. Structural comparisons of virion proteins have shed light on emergence of many viral lineages (Krupovic and Koonin 2017), but traditional phylogenetic methods have been more difficult to employ. In contrast to smaller viruses, large DNA viruses encode several useful phylogenetic markers that generally provide a cohesive phylogenetic signal, and as such they may afford an opportunity to examine viral evolutionary origins in more detail. For example, phylogenomic studies of the *Nucleocytoviricota* have found that trees made with the family B DNA polymerase (PolB), multi-subunit RNA polymerase (RNAP), and mRNA-capping enzyme generally provide consistent evolutionary relationships in this phylum (Aylward et al. 2021). Other proteins, such as the major capsid protein, D5 helicase/primase, ribonucleotide reductase, A32 packaging ATPase, and some transcription factors are also useful phylogenetic markers when inferring relationships within the *Nucleocytoviricota*, but their sparse distribution in lineages outside the phylum make them less useful for analysis of the evolutionary origins of this group. Similarly, Mirusviruses also typically encode PolB and RNAP homologs that have consistent phylogenetic signals (Gaïa et al. 2023), though less phylogenomic work has been performed on this group owing to their recent discovery. In this study, we sought to examine the evolutionary origins of the *Nucleocytoviricota* in the context of eukaryotic diversity through phylogenetic analysis of the PolB, RNAP, and mRNA-capping enzymes encoded by this group. Several previous studies have performed phylogenetic analysis on these enzymes (Yutin and Koonin 2009; Takemura et al. 2015; Yoshikawa et al. 2019; Guglielmini et al. 2019), and our goal was to provide an updated analysis with a broader sampling of both viral and cellular lineages.

Most phylogenomic studies of eukaryotes have concluded that LECA had a complex cellular architecture and a genome that encoded RNAP I, II, and III as well as the four family B DNA polymerases Polα, Polζ, Polε, and Polδ (Gabaldón 2021; Richards et al. 2024). This complexity is in stark contrast to bacteria and archaea, which encode only one RNAP complex and typically have a smaller set of polymerases. Some of these eukaryotic enzymes bear signatures of ancient evolutionary links to each other, suggesting that they may be the product of gene duplications that took place prior to the emergence of LECA. For example, eukaryotic RNAP I, II, and III all form distinct clades, while Polα, Polζ, and Polδ are placed in the same region of our PolB tree (sometimes referred to broadly as the “Polδ clade”). By examining the placement of viral enzymes relative to their eukaryotic homologs, it may be possible to ascertain the timing at which certain viral lineages emerged. If a lineage of viruses acquired an enzyme from eukaryotes after the emergence of LECA, we would expect that the clade of viral enzymes would be nested within a corresponding clade of eukaryotes (i.e., viral RNAP nested within any of the three RNAP clades, or PolB nested within any of the four PolB clades). If the lineage of viruses acquired this enzyme prior to the emergence of LECA, however, we would expect that the viral clade would be placed outside of one of these well-defined clades of eukaryotic enzymes.

In our analysis, nucleocytovirus PolB, RNAP, and mRNA-capping enzymes are not nested within eukaryotic clades that can be traced to LECA, suggesting that they potentially emerged through pre-LECA gene transfer events. In the case of PolB, most nucleocytovirus sequences form a sister clade to eukaryotic Polδ, while two medusavirus proteins are basal-branching relative to these clades. Moreover, herpesviruses and mirusviruses encode PolBs that are placed near but outside the eukaryote/nucleocytovirus clade. Assuming that the root of the PolB tree can be placed somewhere in the archaea, this would suggest that several distinct viral lineages acquired their PolBs prior the emergence of current Polδ clade (i.e., prior to the emergence of LECA). This potentially occurred in multiple host-to-virus gene transfer events. In the scenario in which eukaryotic Polδ, Polα, and Polζ all emerged from ancient gene duplication events, these gene transfers with viruses would have likely occurred afterwards due to the placement of viral clades surrounding the EukPolδ clade.

Similarly, nucleocytovirus RNAP forms a clade with mirusviruses that is proximal to, but not nested within, eukaryotic RNAP II. A previous study reported a similar relationship, albeit without mirusviruses (Guglielmini et al. 2019). The RNAP tree can be more confidently rooted owing to the presence of bacteria in this tree. This topology is consistent with the potential duplication of an ancestral RNAP into type I, II, and III prior to the viral acquisition of type II. The tree of mRNA-capping enzymes is the most difficult to interpret owing to the challenges of rooting this tree, but the clade of nucleocytovirus enzymes is not nested within one of eukaryotic homologs, which would be expected under a scenario of post-LECA host-to-virus gene transfer.

**Fig. 4:**
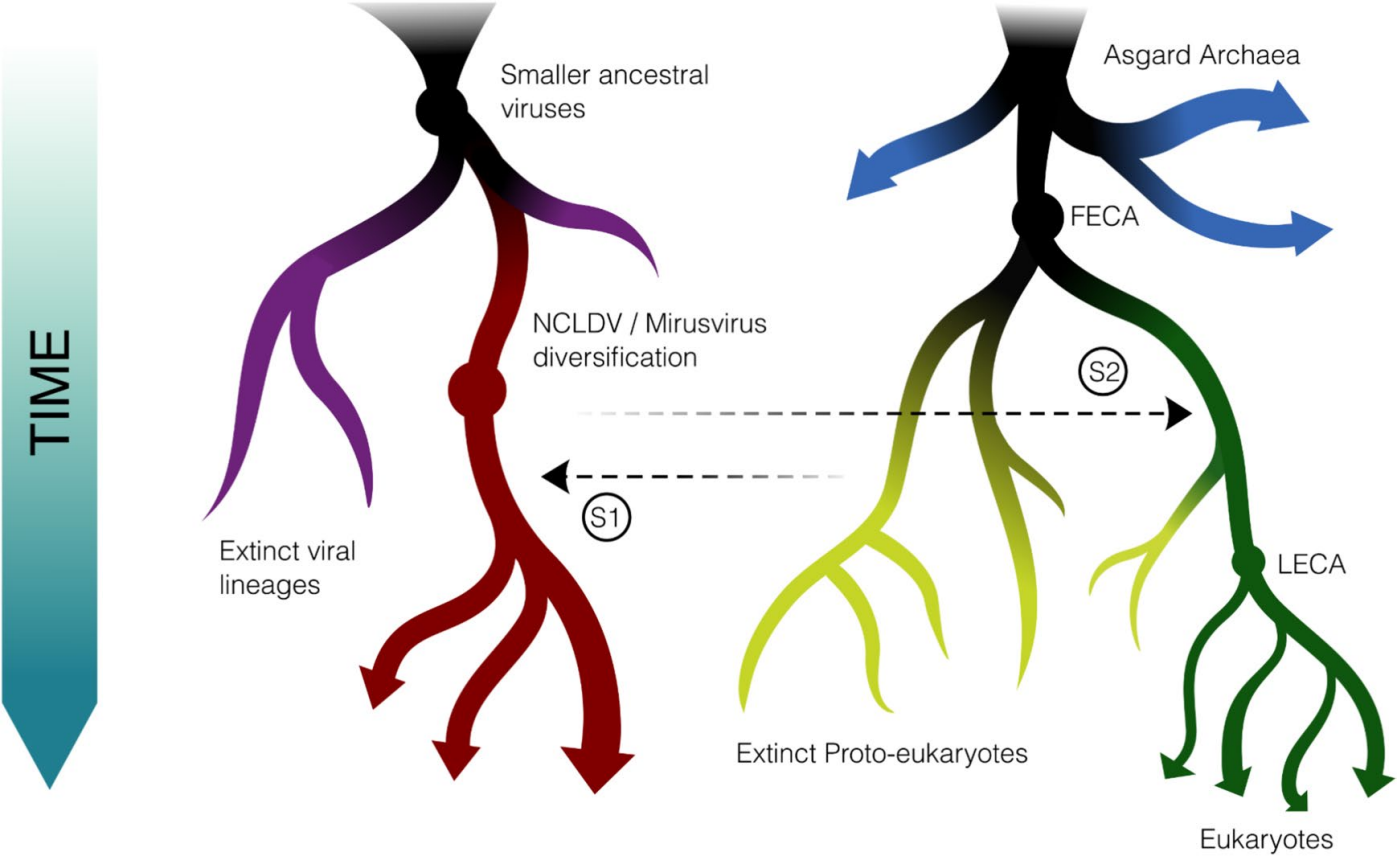
Schematic of possible evolutionary scenarios that would lead to the nested placement of core eukaryotic genes within broader clades of viruses. In scenario 1 (S1), viruses acquire core machinery from proto-eukaryotic lineages that subsequently go extinct, In scenario 2 (S2), virus-to-eukaryotic gene transfer takes place prior to the emergence of LECA

We propose that two evolutionary scenarios could explain the evolutionary patterns revealed in the PolB, RNAP, and mRNA-capping enzyme trees. The first involves host-to-virus gene transfer that occurred prior to the emergence of LECA, while the second involves a possible virus-to-host gene transfer from a viral progenitor (scenarios 1 and 2, respectively, in Fig. 4). According to the first scenario, nucleocytoviruses acquired the machinery for DNA replication, transcription, and mRNA capping prior to the emergence of LECA, consistent with the placement of these viral enzymes outside of any clade that can be traced to LECA. This would potentially involve transfer from proto-eukaryotic lineages that either subsequently went extinct or have not yet been discovered. Some of these transfers may have even occurred several times independently, consistent with the evidence that host-to-virus transfers tend to be several times more common than the reverse (Irwin et al. 2022). Indeed, the curious placement of the medusavirus PolBs suggest that this lineage may have acquired their polymerase independently. These pre-LECA gene transfers could explain why eukaryotic Polδ appears to be placed within a broader viral clade that includes nucleocytoviruses, mirusviruses, and herpesviruses. Importantly, this scenario is consistent with the general hypothesis of “gene accretion” that was originally proposed for nucleocytoviruses (Iyer et al. 2006).

In this first scenario of pre-LECA host-to-virus gene transfer, one may legitimately ask why these host-to-virus transfers appear to be proximal to eukaryotic Polδ and not Polα, Polζ, or Polε. Given that Polδ plays a key role in processive DNA replication in eukaryotes, it is potentially more likely that this enzyme would have been acquired by viruses rather than the related Polα and Polζ homologs, which play roles in initiation and repair and may be less easily co-opted for viral genome synthesis. Similarly, due to the role of RNAP II in mRNA transcription, it is possible that this enzyme could be more easily co-opted for viral gene expression compared to RNAP I and III, which would explain why viruses obtained a progenitor to RNAP II. The preferential recruitment of viral enzymes that are best suited for viral replicative processes may, therefore, explain these patterns.

In the second scenario, some eukaryotic enzymes may have been acquired from viruses. A viral origin of these enzymes in eukaryotes remains a possibility, in principle, and this has been previously postulated (Villarreal and DeFilippis 2000; Takemura 2001; Bell 2001). A viral origin of Polδ would appear contrary to the hypothesis that this enzyme arose, together with Polζ and Polα, from a series of ancient duplications, and similarly a viral origin of RNAP II would seem to contradict the scenario in which RNAP I, II, and III arose from ancient duplications. We, therefore, favor the first scenario to explain our results, but we anticipate that further phylogenetic analyses and discovery of new viral lineages will help to clarify these deep evolutionary links.

A defining feature of the *Nucleocytoviricota* is the virus factory, also called the viroplasm, which is a complex intracellular structure that forms during infection and is the location of DNA synthesis, viral transcription, and virion morphogenesis. Although not all members of the *Nucleocytoviricota* form virus factories during infection, it is a prevalent feature of giant viruses spanning all six orders and two classes of this phylum and is most likely a trait that was present in their last common ancestor. Central importance has been placed on virus factories in the biology of nucleocytoviruses (Claverie 2006), and assessing the origin of this structure is key to understanding the origin of the *Nucleocytoviricota*. Proper functioning of the virus factory would necessarily require a viral mechanism for DNA polymerization, transcription, and mRNA processing, and it is, therefore, interesting to note that viral enzymes involved in these processes exhibit ancient evolutionary origins. We propose that the most parsimonious scenario that explains the deep-branching topologies of the family B DNA polymerase, DNA sliding clamp, and multimeric RNA polymerase, and mRNA-capping machinery is pre-LECA origin of the virus factory. Recent evidence suggests that stem eukaryotes diversified for a long period of time prior to the emergence of LECA (Brocks et al. 2023), and it is, therefore, plausible that these stem eukaryotes housed a complex array of viruses that gave rise to the modern *Nucleocytoviricota* (Krupovic et al. 2023). It is possible that the virus factory was a key innovation that potentially allowed early nucleocytoviruses to evade host defenses by creating a physical barrier between the host cytoplasm and the site of viral replication and transcription, leading to an adaptive radiation of this phylum. The nucleocytovirus virus factory is structurally analogous to the “phage nucleus” generated by some large bacteriophages during infection (Chaikeeratisak et al. 2017), and is, therefore, a mechanism employed by several distinct lineages in the virosphere.

## Materials and Methods

### Dataset Compilation

We compiled a set of high-quality bacterial, archeal, and eukaryotic and viral genomes for subsequent phylogenetic analyses. For eukaryotic genomes, we used all genomes available on the eggNOG v5.0 database (Huerta-Cepas et al. 2019). To increase the representation of unicellular eukaryotes, we also included seven complete or chromosome-level genomes of protists available on the National Center for Biotechnology Information (NCBI) databases (Sayers et al. 2024) as of October 8, 2021. For bacterial and archaeal genomes, we retrieved genomes from the Genome Taxonomy Database (GTDB, v95) (Chaumeil et al. 2019). To enrich our database in Asgard archaeal genomes, we also included genomes from this group that were reported in a recent large-scale comparative genomic study (Liu et al. 2021) that were not already present in the GTDB. For viral lineages, we focused on members of the *Herpesvirales*, *Nucleocytoviricota* (i.e., “giant viruses”), and the recently discovered phylum *Mirusviricota*. We used complete herpesvirus genomes available in NCBI as of July 2023, all nucleocytovirus genomes available in the Giant Virus Database (GVDB) (https://faylward.github.io/GVDB/) (Aylward et al. 2021), and all mirusvirus genomes published in a recent study (Gaïa et al. 2023). For the PolB analysis, we also considered including sequences derived from other viral groups that encode this enzyme, such as some tailed phages (class *Caudoviricetes*), adenoviruses, baculoviruses, polinton-like viruses, virophages, and some recently discovered viruses of Asgard archaea (Rambo et al. 2022). In initial diagnostic trees that we constructed for PolB (see methods below) these sequences formed long branches that clustered with archaeal PolB 1, 2, and 3 clades, and we, therefore, removed them from our final analysis on the grounds that these long branches could compromise the overall topology of the tree. Moreover, the PolBs from most of these viral groups are protein primed (not processive), and therefore, not as relevant to our analysis given the focus of our work on processive DNA polymerase evolution. For eukaryotic genomes, we used protein predictions already available on EggNOG v. 5.0, and for all other taxa we predicted proteins using Prodigal v. 2.6.3 with default parameters (Hyatt et al. 2010).

### Sampling of Taxa

Highly biased taxon sampling can adversely affect phylogenetic inference (Martinez-Gutierrez and Aylward 2021). We, therefore, sought to balance the number of different lineages used in our subsequent phylogenetic analyses by subsampling groups of over-represented lineages, which for our purposes were bacteria, archaea, plant, metazoans, fungi, and giant viruses. For bacteria and archaea, we chose high-quality representative genomes from each class in the GTDB to include using a methodology described previously. For eukaryotes, we manually selected a subset of 127 genomes to include in order to remove the overabundance of genomes from the Fungi, Opisthokonta, and Viridiplantae lineages in the EggNOG database, and we added 7 complete or chromosome-level genomes of protist lineages from the NCBI. For nucleocytoviruses, we down-sampled the full set of 1381 genomes in the GVDB to 343 by including only genus-level representatives from the taxonomy available in this database. For this down-sampling, we chose the genome of the genus-level representative with the highest N50 contig length. We did not down-sample mirusviruses and herpesviruses because relatively few genomes from these lineages were already available. After this down-sampling, we arrived at a genome set that included 127 eukaryotes, 279 archaea, 230 bacteria, 343 nucleocytoviruses, 111 mirusviruses, and 113 herpesviruses. These genomes were a starting point for phylogenetic inference of all trees in our study, and most of the trees that we subsequently analyzed did not include all of these taxa because some lineages lack certain proteins (e.g., most bacteria do not encode family B DNA polymerases). A full list of all genomes used is available in https://zenodo.org/records/10956246 and Supplementary Table 1.

### Dataset Curation and Quality Check

For prediction of PolB, PCNA,and mRNA-capping enzyme homologs in our genome set, we used a custom python script that uses the hmmsearch command in HMMER3 (Eddy 2011) (see Code Availability section). For Hidden Markov Model (HMM) references we used PolB and PCNA models from Pfam v. 32.0 (Mistry et al. 2021) (accessions PF00136 and PF00705 respectively). For multi-subunit RNA polymerase (RNAP), we used the markerfinder_v2.py script to both identify homologs of the beta and betaprime subunit of RNAP and then concatenate them together into a single alignment. For eukaryotes, we did this by matching to custom HMMs that we designed for these subunits in RNAP I, II, and III. For identification of beta and betaprime RNAP subunits in bacteria, archaea, and viruses, we used the COG0085 and COG0086 HMMs designed previously (Martinez-Gutierrez and Aylward 2021). For all trees, prior to alignment we first de-replicated nearly-identical sequences using CD-HIT version 4.8.1(Fu et al. 2012). For PolB trees, we also removed all sequences < 650 aa on the grounds that these were likely truncated or erroneously predicted. For the RNAP tree, we did not include taxa in the analysis unless both the beta and betaprime subunit could be identified and included in the alignment.

### Phylogenetic Tree Reconstruction and Benchmarking

For all alignments we used Muscle5 (Edgar 2022) (parameters “-super5” for input sequences), which has recently been shown to substantially improve multi-sequence alignment compared to previous methods. For RNAP specifically, we used a custom script merge_and_align.py that uses Muscle5 algorithm to align and then concatenate the RNAP protein sequences (see code availability section). We trimmed the alignments with trimAl v1.4. rev15 (Capella-Gutiérrez et al. 2009) (parameter -gt 0.1 but see below for alternative trimming strategies). We manually inspected all alignments with AliView (Larsson 2014) and removed sequences with long, continuous gaps that may hinder phylogenetic inference. In these cases, alignment was then re-performed, and the alignments were inspected again. In the case of PolB, we inspected the untrimmed alignments and found some long insertions in some sequences that correspond to inteins, but we confirmed that these were removed by subsequent trimming steps.

For all the gene trees (PolB, RNAP, and PCNA), we initially constructed diagnostic phylogenetic trees using IQ-TREE v1.6.12 (Nguyen et al. 2015) with the option -bb 1000 to generate 1000 ultrafast bootstraps (Minh et al. 2013), -m MFP to determine the best-fit model (Kalyaanamoorthy et al. 2017), -nt AUTO and –runs 5 to select the highest likelihood tree. These initial trees were inspected, and long branches that represent rogue taxa or low-quality sequences were removed (< 10 sequences from each tree) prior to re-alignment. Moreover, upon inspecting the initial diagnostic trees, we noticed that several large clades of giant viruses and archaea were present, and we randomly down-sampled these clades by 20% using the seqtk subseq function to lessen the computational burden and further prevent biased taxon sampling across the tree. We also noticed that poxviruses had unstable placement in our diagnostic trees, consistent with previous findings (Guglielmini et al. 2019), and we, therefore, removed this lineage from further analyses. After rogue taxa removal and the last round of subsampling, alignment and trimming procedures were run again.

Once the final alignment was obtained, we then reconstructed maximum likelihood phylogenetic trees using IQ-TREE (parameters -bb 1000, -m MFP, -nt AUTO, –runs 5). The LG+F+R10 model was selected as best-fit substitution model based on Bayesian Information Criterion (BIC) for the PolB and RNAP tree, while LG+F+R7 was chosen as best fit for the PCNA tree by ModelFinder (-m MFP). Because amino acid substitution rates likely vary across alignments, we also inferred trees using complex models (C-models) that have different substitution matrices for every position in the alignment(Quang et al. 2008) (LG+C60+F+G). We then compared our models from the -MFP option to the most complex C60 model. Although the trees inferred with the -MFP option generally had lower BICs, we still examined the trees inferred with a complex model (LG+C60+F+G) to assess any differences in topology that could be detected using the different methods. mRNA capping enzyme trees were also reconstructed using the same alignment and trimming strategies and trees were inferred using LG+F+R10 model.

### Further Phylogenetic Tree Validation

We performed several tests to examine how alignment trimming severity, removal of fast-evolving sites, and taxon sampling affected our phylogenetic inference. To examine if different trimming methods could impact the topology of our PolB or RNAP trees, we re-made these trees using more stringent levels of alignment trimming (see Supplementary Figs. 6 and 7). Our original trimming strategy was to remove all sites with > 90% gaps (-gt 0.1 option in trimAl), and so for more stringent trimming, we removed all sites with 50% or more gaps (-gt 0.5 parameter) or by using the automated trimming stringency (-automated1 option). For PolB, this resulted in alignment lengths of 867aa (for -gt 0.5) and 351aa (for -automated1) compared to 1417aa for the primary alignment. For our concatenated RNAP alignment, this resulted in alignment lengths of 2420aa (for -gt 0.5) and 1117aa (for -automated1) compared to 3812aa for the primary alignment that we used in our analysis. After generating these alternatively trimmed alignments, we inferred phylogenies in IQ-TREE using the same LG+F+R10 substitution model as determined by ModelFinder.

Taxon sampling has been shown to impact phylogenetic tree inference, and we, therefore, sought to examine if the topology of our trees were consistent when using a smaller set of taxa. To test the effect of taxon down-sampling, we down-sampled the PolB and RNAP protein sequences by ~ 50% to 375 sequences for PolB and 517 sequences for RNAP, while keeping the overall proportion of cellular and viral groups consistent (see Extended Data Fig. 3). We then generated alignments with Muscle5, used the same alignment QC procedure described for our original trees, and generated trees in IQ-TREE using the LG+F+R10 substitution model.

Lastly, we sought to examine if the removal of fast-evolving sites would alter the topology of our trees (see Extended Data Fig. 4). It has been suggested that the removal of fast-evolving sites helps increase the signal-to-noise ratio in phylogenetic inference (Rodríguez-Ezpeleta et al. 2007), although a recent study has indicated that fast-evolving sites are informative for tree building (Rangel and Fournier 2023). We, therefore, inferred site-specific evolutionary rates from our trimmed PolB and RNAP primary alignments using the -wsr parameter in IQ-TREE v1.6.12 (Nguyen et al. 2015). This produced ten different rate categories, which we then sequentially removed before inferring trees with the LG+F+R10 model.

## Supplementary Information

Below is the link to the electronic supplementary material.

Supplementary file1 (DOCX 1377 KB)

Supplementary file2 (XLSX 209 KB)

Supplementary file3 (XLSX 5 KB)

## Data Availability

All genomes and alignments used in this study can be found here: https://zenodo.org/records/10956246. All phylogenetic trees are available on interactive Tree of Life (iTOL): https://itol.embl.de/shared/15ttJikbnoVmi and https://itol.embl.de/shared/1l6saIRHqS5eY.

## Code Availability

PolB and PCNA homolog identification was done using the custom python script hmmsearch_wrapper: https://github.com/sangitakarki/hmmsearch_wrapper. RNAP subunits were identified and concatenated alignments were generated using markerfinder-euk: https://github.com/faylward/markerfinder-euk.
