# Supplementary material for: Ancient Host-Virus Gene Transfer Hints at a Diverse Pre-LECA Virosphere
Source: J Mol Evol. 2025 Apr 29;93(3):295–305. doi: 10.1007/s00239-025-10246-8 (PMC12198294; doi:10.1007/s00239-025-10246-8)
Supplement: Supplementary file 1 — Supplementary file1 (DOCX 1377 KB) [file 239_2025_10246_MOESM1_ESM.docx]

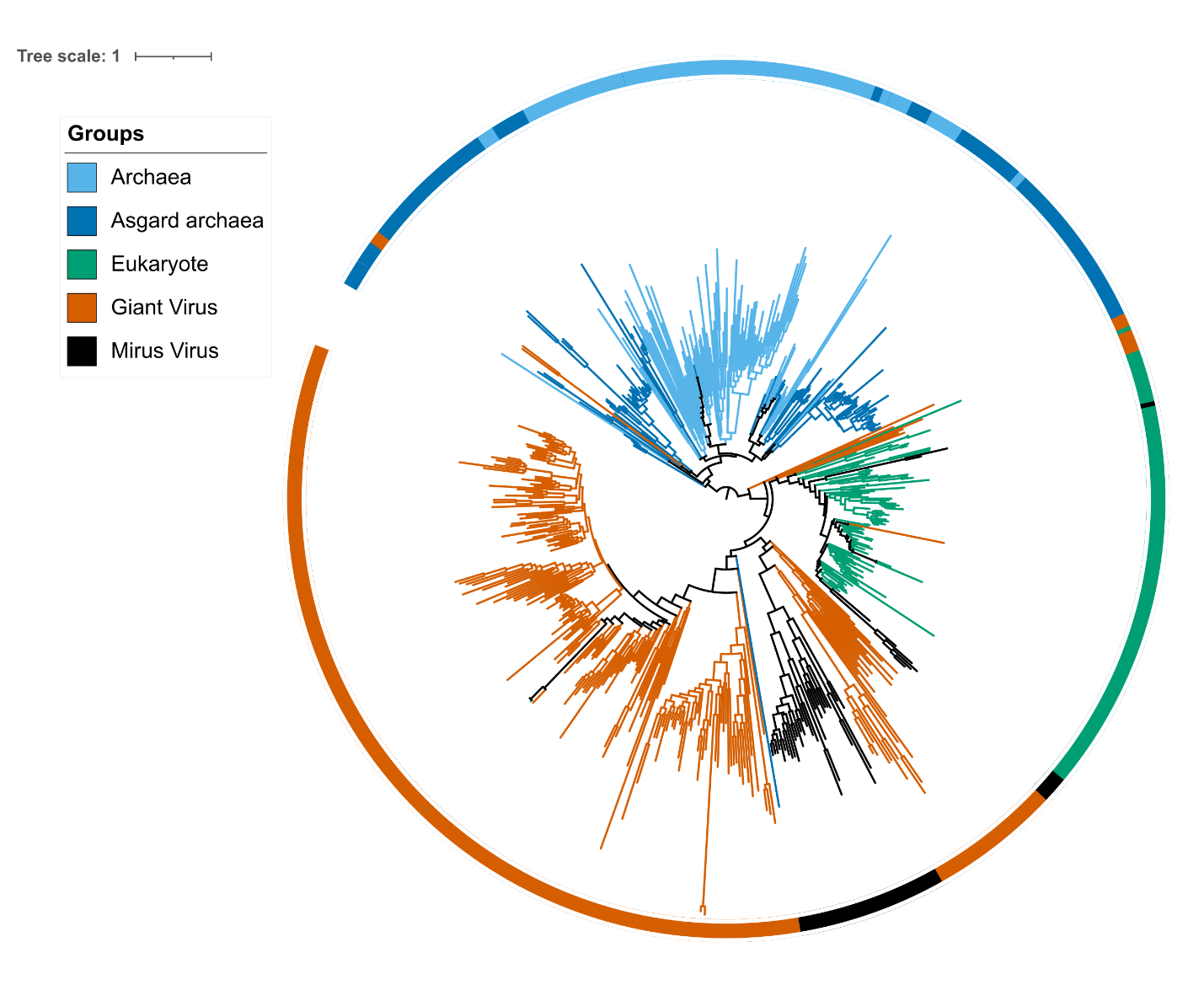


**Fig. 1** | Phylogenetic tree for DNA Sliding Clamp (PCNA) for 606 sequences (resulting in total alignment length of 289 sites). Maximum-likelihood analysis was performed using IQ-TREE under a complex model (LG+C60+F+G). The tree is rooted within an archaeal group. The tree shows eukaryotic sliding clamp clustered within the viral group.


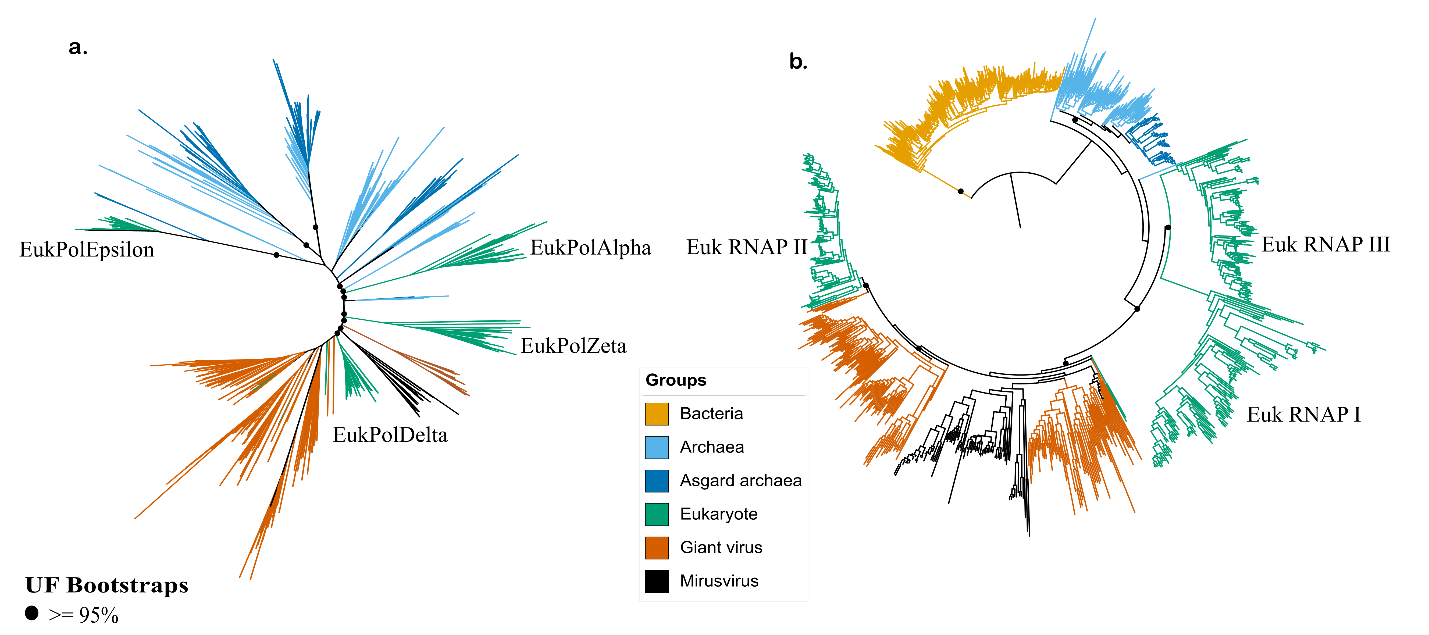


**Fig. 2** | Phylogenetic tree for DNA Polymerase B and RNA Polymerase. The main text figures show trees with complex model while these trees are inferred using regular model. The trees were inferred using the LG+F+R10 model that was chosen as best fit by ModelFinder (-MFP). a) DNA Polymerase B tree is unrooted while b) RNA Polymerase is rooted within bacteria. Dots on the main nodes represent ultrafast bootstrap support.


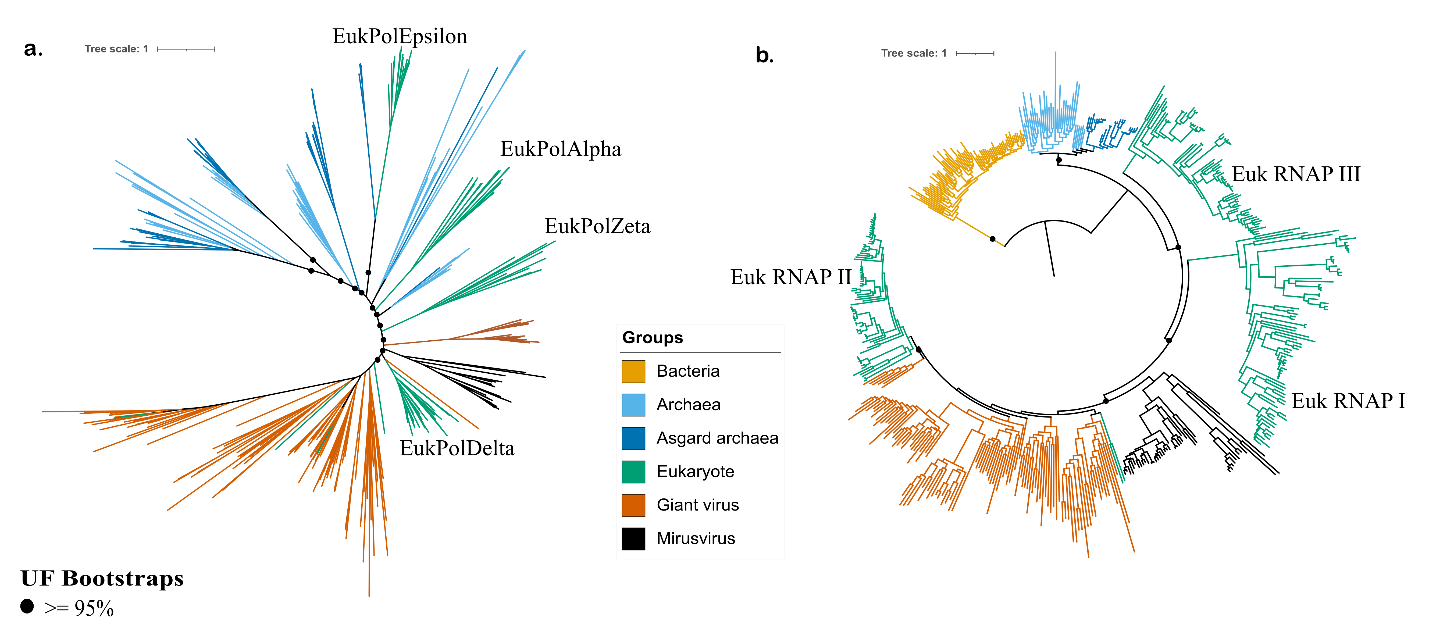


**Fig. 3** | Phylogenetic tree for DNA Polymerase B and RNA Polymerase reduced sets (375 PolB sequences and 517 RNAP sequences vs. 957 PolB sequences and 1017 RNAP sequences previously). The trees were inferred using the LG +F+R10 model. a) DNA Polymerase B tree is unrooted while b) RNA Polymerase is rooted within bacteria. Dots on the main nodes represent ultrafast bootstrap support.


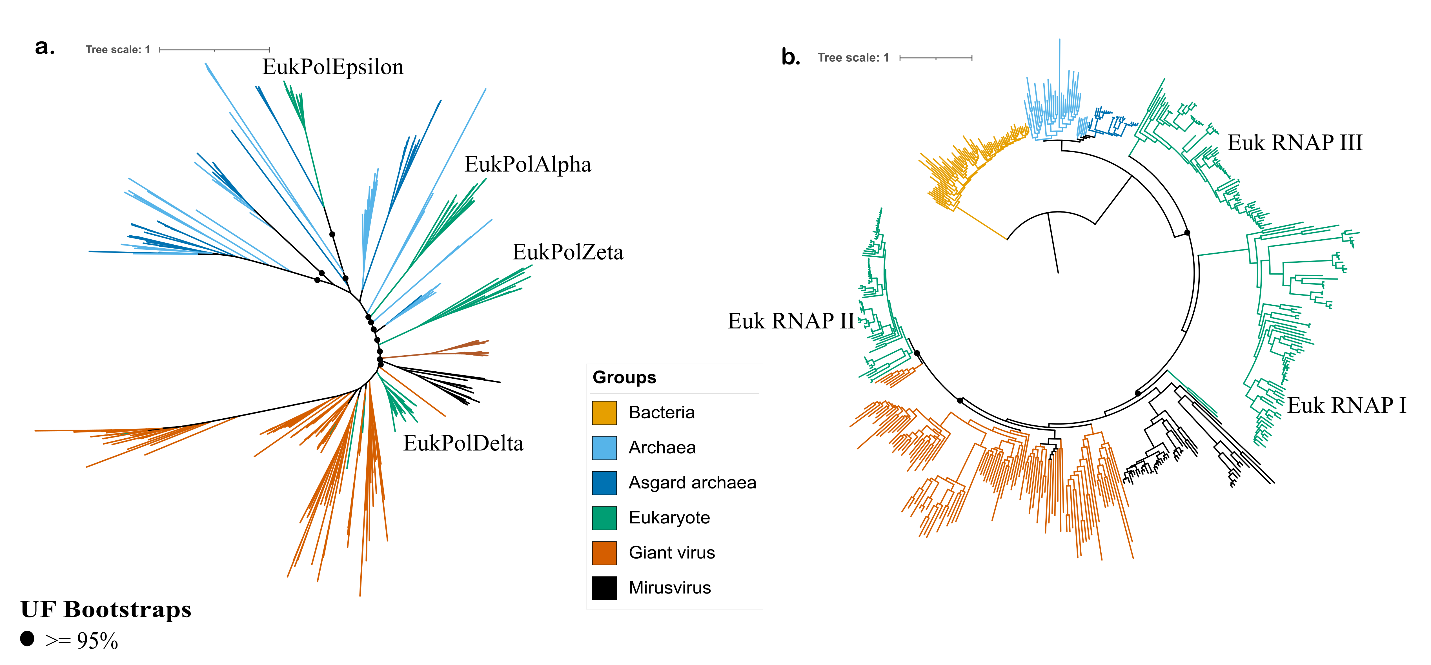


**Fig. 4** | Representative phylogenetic tree after removing 30% of the fast-evolving sites (0-slowest, 10-fastest). The topology is still intact and well supported for viral derives. The trees were inferred using the LG +F+R10 model. a) DNA Polymerase B tree is unrooted while b) RNA Polymerase is rooted within bacteria. Dots on the main nodes represent ultrafast bootstrap support.


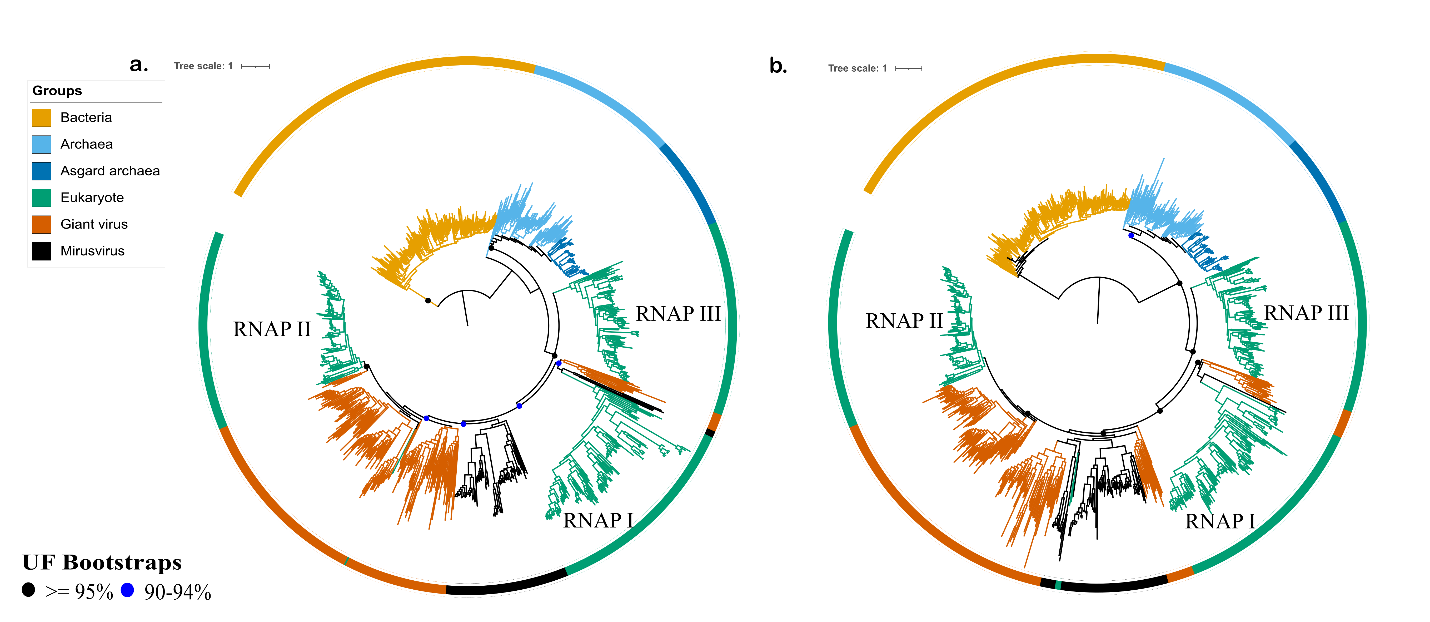


**Fig. 5** | Individual phylogenetic tree for Beta and beta prime subunits of RNA polymerase. The trees were inferred using the LG +F+R10 model. Both trees are rooted within Bacteria. a) RNA polymerase Beta subunit. b) RNA polymerase Beta prime subunit. Dots on the main nodes represent ultrafast bootstrap support.


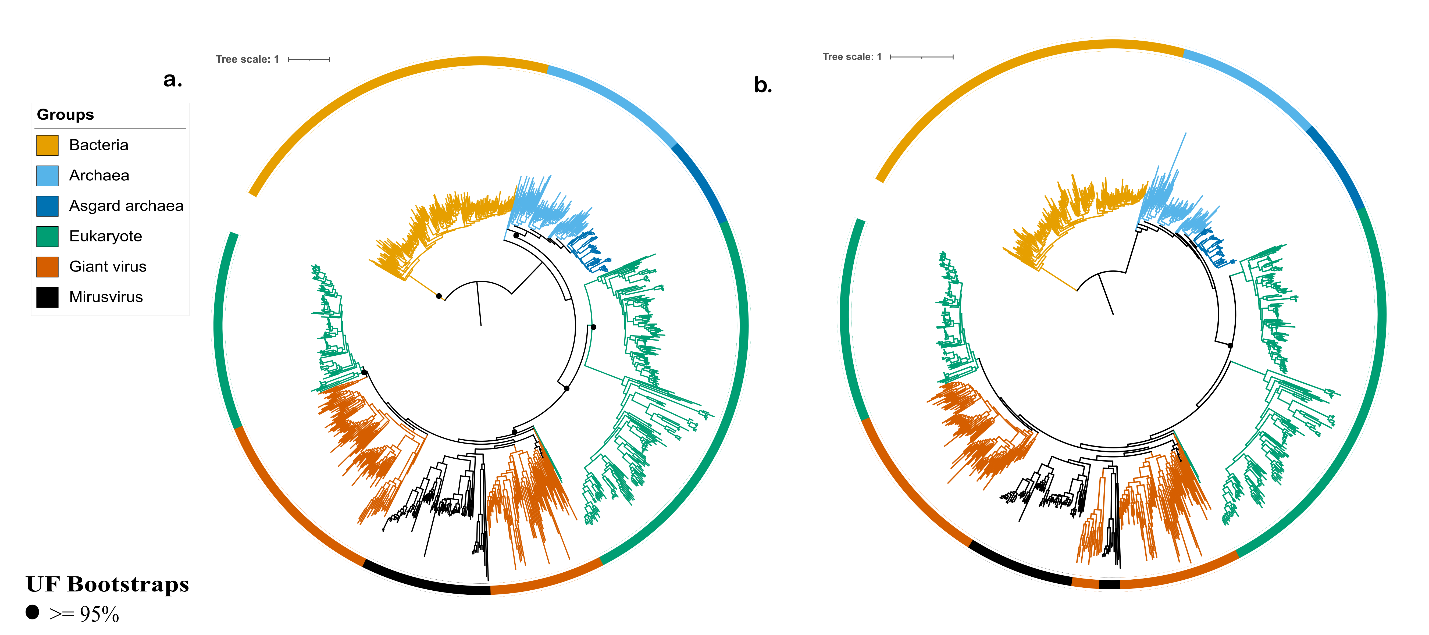


**Fig. 6**| Phylogenetic tree for concatenated RNAP tree using different trimming parameters. a) trimmed parameter -gt 0.5 b) trimmed parameter -gt automated1. Both trees were inferred using the LG +F+R10 model. Both trees are rooted within Bacteria. Dots on the main nodes represent ultrafast bootstrap support.


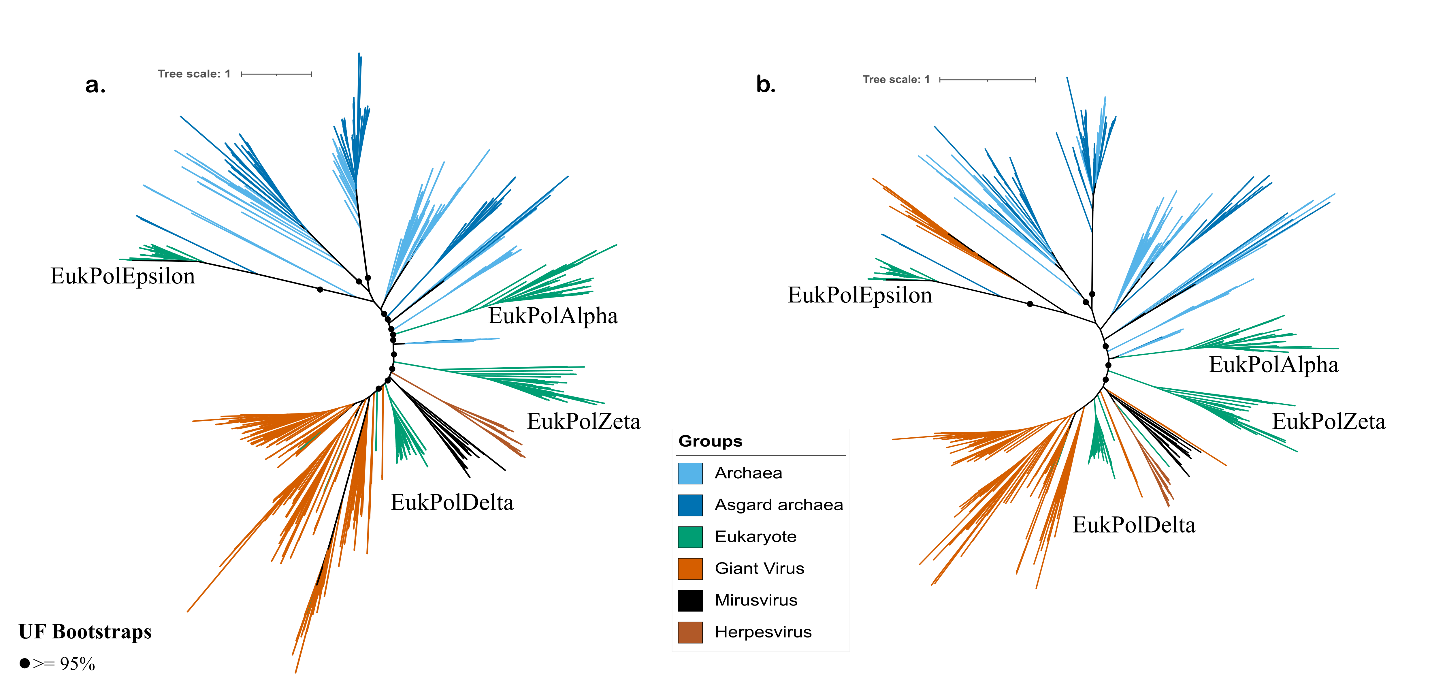


**Fig. 7** | PolB phylogenetic tree using different trimming parameters. a) trimmed parameter -gt 0.5 b) trimmed parameter -gt automated1. Both trees were inferred using the LG +F+R10 model. Dots on the main nodes represent ultrafast bootstrap support.
